# Analysis of viral pneumonia and risk factors associated with severity of influenza virus infection in hospitalized patients from 2012 to 2016

**DOI:** 10.1186/s12879-024-09173-8

**Published:** 2024-03-12

**Authors:** M. I. Fullana Barceló, F. Artigues Serra, A. R. Millan Pons, J. Asensio Rodriguez, A. Ferre Beltran, M. del Carmen Lopez Bilbao, J. Reina Prieto, M. Riera Jaume

**Affiliations:** 1 Internal Medicine Department, Infectious Diseases Section, Hospital Universitari Son Espases, Valldemossa Road 79, Palma de Mallorca, 07010 Spain; 2 IdISBa estadistic and methodological support, Hospital Universitari Son Espases, Palma de Mallorca, Spain; 3 Preventive Medicine Department, Hospital Universitari Son Espases, Palma de Mallorca, Spain; 4 Microbiological Department, Hospital Universitari Son Espases, Palma de Mallorca, Spain; 5 IdISBa– Fundació Institut d’Investigació Sanitària Illes Balears, Palma de Mallorca, Spain

**Keywords:** Influenza risk factors, Coinfection, Influenza vaccination

## Abstract

**Background:**

Influenza viruses cause pneumonia in approximately one-third of cases, and pneumonia is an important cause of death. The aim was to identify risk factors associated with severity and those that could predict the development of pneumonia.

**Methods:**

This retrospective, observational study included all adult patients with confirmed influenza virus infection admitted to Son Espases University Hospital during four influenza seasons in Spain (October to May) from to 2012–2016.

**Results:**

Overall, 666 patients with laboratory-confirmed influenza were included, 93 (14%) of which were severe; 73 (10.9%) were admitted to Intensive Care Unit (ICU), 39 (5.8%) died, and 185 (27.7%) developed pneumonia. Compared to less severe cases, patients with severe disease: were less vaccinated (40% vs. 28%, *p* = 0.021); presented with more confusion (26.9% vs. 6.8%), were more hypoxemic (Horowitz index (PaO_2_/FiO_2_) 261 vs. 280), had higher C-reactive protein (CRP) (12.3 vs. 4.0), had more coinfections (26.8% vs. 6.3%) and had more pleural effusion (14% vs. 2.6%) (last six all *p* < 0.001). Risk factors significantly associated with severity were pneumonia [OR (95% CI) = 4.14 (2.4–7.16)], history of heart disease (1.84, 1.03–3.28), and confusion at admission (4.99, 2.55–9.74). Influenza vaccination was protective (0.53, 0.28–0.98). Compared to those without pneumonia, the pneumonia group had higher CRP (11.3 vs. 4.0, *p* < 0.001), lower oxygen saturation (92% vs. 94%, *p* < 0.001), were more hypoxic (PaO_2_/FiO_2_ 266 vs. 281, *p* < 0.001), and incurred more mechanical ventilation, septic shock, admission to the ICU, and deaths (all four *p* < 0.001). Higher CRP and lower oxygen saturation were independent variables for predicting the development of pneumonia.

**Conclusions:**

Pneumonia, history of heart disease, confusion and no influenza vaccination were independent variables to present complications in patients admitted with influenza infection.

## Background

Influenza virus infection causes pneumonia in approximately one-third of patients [1]. In addition, viruses are the only cause of pneumonia in up to 20% of influenza cases [2]. It is widely known that viral infection predisposes patients to secondary bacterial infection, which is one of the complications associated with higher mortality [3]. Despite improvements in viral identification with the introduction of real-time polymerase chain reaction (PCR) in 2008 [2], differentiating viral pneumonia from secondary bacterial pneumonia remains challenging [4, 5].

Viral pneumonia has an insidious course, affecting older people and patients with a high number of comorbidities, as well as long-term care institutionalized persons [6, 7]. Regarding radiological features, viral pneumonias often show interstitial infiltration, whereas bacterial pneumonias more commonly show alveolar infiltration [8]. Little information existed about factors associated with poor prognosis in viral pneumonia, especially before the COVID-19 pandemic; however, there has been increasing interest in establishing pneumonia etiology and differentiating viral pneumonia from secondary bacterial pneumonia since. In addition, there are few publications on influenza virus complications outside of the intensive care unit (ICU).

The main objective of this study was to determine risk factors related to severe infection, defined as death or ICU admission, in patients with a diagnosis of influenza virus infection at the time of hospital admission. A secondary objective was to describe the variables that can predict the development of pneumonia in these patients with influenza virus infection.

## Methods

This retrospective observational study included adult patients with laboratory-confirmed influenza virus infection and those diagnosed during admission who were admitted to Son Espases University Hospital during influenza seasons 2012–2013 to 2015–2016. Clinical data were collected from medical reports from the moment of the admission. The period of influenza surveillance in Spain ranges from epidemiological week 40 of one year to epidemiological week 20 of the following year (October to May). All adult patients (aged > 18 years) with laboratory-confirmed influenza virus infection were included. A case of confirmed influenza was defined as adult patients admitted for influenza syndrome (onset of systemic symptoms such as fever, malaise, myalgia, and respiratory symptoms including cough, sore throat, shortness of breath, and absence of other suspected diagnosis) confirmed by PCR of ribonucleic acid detection in respiratory samples processed in the microbiology laboratory of Son Espases University Hospital. A nosocomial case was defined as a case in patients with symptoms onset 2 days after admission, when the admission was for reasons other than respiratory symptoms. Most of the complementary tests were performed on patients are those performed upon the patient’s arrival to the emergency room. All chest x-rays have been reviewed and compared with the report at the disharge report. The presence of interstitial infiltrates on chest radiography in these patients with associated symptoms was considered viral pneumonia, after rulig out other causes of interstitial involvement such as heart failure. A coinfection was defined as having other bacterial and/or viral infection along with confirmed influenza, and a positive complementary test for another microorganism (blood culture, sputum culture or other respiratory sample) has been considered coinfection. A severe or complicated case was defined as the need for admission to the ICU or death.

Qualitative variables are described as frequencies and percentages, whereas quantitative variables are described as medians and interquartile ranges. The difference between patients with severe and non-severe infections was analyzed using the Mann-Whitney U test for quantitative variables and the chi-square test or Fisher test for qualitative variables. Once significant differences were reported, simple and adjusted logistic regression analysis was performed using the backward method to avoid confounding factors and the adjusted effects for the remaining variables were obtained. The independent variables included were age, medical history including smoking, obesity, history of cardiac disease, diabetes mellitus, chronic kindey disease and vaccination status, and most important symptoms and signs most important to assess upon arrival of the patient as dyspnea, state of confusion, systolic pressure less than 90 mmHg, Oxygen saturation less than 93%, arterial oxygen partial pressure to fractional inspired oxygen ratio (Horowitz index or PaO_2_/FiO_2_) less than 300 and the presence of pneumonia. The same analysis was repeated to study the development of pneumonia, and the independent variables included were female sex, age, and symptoms and signs as fever, myalgias, dyspnoea, confusion, heart rate, oxygen saturation, PaO_2_/FiO_2_, urea levels, C-reactive protein and albumin levels. Statistical significance was set at *p* value < 0.05. Statistical analysis was performed using the Methodology and Statistics Support Platform and the Balearic Islands Sanitary Investigation Institute. The statistical software used was IBM SPSS version 26.

## Results

A total of 666 patients were admitted to our hospital with influenza virus infection between 2012 and 2016, 93 (14%) of whom had severe disease: 73 (10.9% of the total) were admitted to the ICU and 39 died (5.8%). Among patients admitted to the ICU, 19 (26%) died. One hundred eighty-five (27.7%) patients were diagnosed with pneumonia and 45 (24%) were admitted to the ICU. The clinical characteristics of all patients included in the study are presented in Table 1.

**Table 1:** Clinical characteristics of all patients included in the study

	Total	N (%)
Sex, (female), n(%)	666	328 (49.2%)
Age (IQR)	666	67 (53–77)
Smoking, n(%)	666	198 (29.7%)
COPD*, n(%)	666	160 (24%)
Pregnancy, n(%)	666	5 (0.8%)
Obesity, n(%)	666	93 (14%)
Cardiac disease, n(%)	666	221 (33.2%)
Chronic kidney disease, n(%)	666	84 (12.6%)
Diabetes, n(%)	666	192 (28%)
Immunosuppression, n(%)	666	
HIV**, n(%)		45 (6.8%)
Malignancy, n(%)		42 (6.3%)
Corticotherapy, n(%)		18 (2.7%)
Others, n(%)		31 (4.7%)
Vaccinated, n(%)	666	258 (38.7%)
Subtype, n(%)	666	
H1N1		242 (36.3%)
H3N2		241 (36.2%)
Influenza A no subtype		33 (5%)
Influenza B		150 (22.5%)
Cough, n(%)		573 (86%)
Fever, n(%)		476 (71.5%)
Myalgia, n(%)		166 (24.9%)
Dyspnea, n(%)	666	446 (67%)
State of confusion, n(%)	666	64 (9.6%)
Heart rate, median (IQR), n total	650	96 (82–109)
Systolic blood pressure [mmHg], median (IQR), n total	655	131 (113–147)
Respiratory frequency, median (IQR), n	176	26 (20–32)
Oxygen saturation [%], median (IQR), n	655	93 (90–96)
Oxygen saturation < 93%, n(%)	655	262 (40%)
Horowitz Index (PaO_2_/FiO_2_^)^, median (IQR), n	527	276 (242–309)
Alveolar-arterial gradient, median (IQR), n	544	46 (38–55)
Alveolar-arterial gradient adjusted for age, median (IQR), n	595	17.4 (14.3–19.7)
Lymphocyte [x10^9^/L] median (IQR), n	666	8155 (6000–11,400)
Haematocrit [%], median (IQR), n	664	39.1 (34.7–42.7)
Platelet [x109/L], median (IQR)	666	201,500 (151,000–258,000)
Creatinine [mg/dl], median (IQR), n	665	0.9 (0.7–1.1)
Urea [mg/d], median (IQR), n	659	36 (28–53)
Albumin [mg/dl], median (IQR), n	474	34.6 (31-37.3)
C-reactive protein [mg/dl], median (IQR), n	510	5.1 (2-11.9)
Pneumonia, n(%)	666	185 (27.8%)
Pleural effusion, n (%)	666	28 (4.2%)
Septic shock, n(%)	666	50 (7.5%)
Mechanical ventilation, n(%)	666	67 (10.1%)
ARDS***, n (%)	666	11 (1.7%)
Intensive care unit admission, n(%)	666	73 (11%)
Death, n(%)	666	39 (5.9%)
ICU admission and death	666	93 (14%)

*COPD: Chronic Obstructive Pulmonary Disease

**HIV:Human Immunodeficiency Virus

*** ARDS: Adult Respiratory Distress Syndrome

Comparative analyses were conducted between severe and non-severe infection cases and data are presented in Table 2. Age was not associated with severity (64 vs. 67 years, *p* = 0.41) and neither was sex (51% male). Tobacco use, obesity, and history of heart disease were associated with a greater number of complications. There were no differences between the groups regarding asthma, COPD, renal failure, diabetes, or immunosuppression. Influenza subtype did not affect the course of infection. Only 28% of patients with severe disease had been vaccinated compared to 40% of the those in the non-severe group (*p* = 0.02). Patients with severe disease presented with confusion, systolic blood pressure < 90 mmHg, high respiratory frequency (more than 20 rpm), and lower PaO_2_/FiO_2_more often than did the non-severe category (261 vs. 280 *p* < 0.001). Patients with severe disease also experienced dyspnea at admission, although the difference was not statistically significant (75% vs. 65%, *p* = 0.06). Regarding blood tests, severe cases showed lymphopenia, hypoalbuminemia, and lower haematocrit levels, whereas creatinine, urea, and C-reactive protein (CRP) levels were higher (4.7 vs. 12.3, *p* < 0.001).

**Table 2:** Comparative analyses between severe and non-severe infection cases

	Non-severe, *N* = 573	Severe (ICU admission and death), *N* = 93	*p*
Sex, (female), n(%)	282 (49.2%)	46 (49.5%)	0.965
Age (IQR)	67 (53–77)	64 (54–75)	0.408
Smoking, n(%)	163 (28.4%)	35 (37.6%)	0.072
COPD *, n(%)	143 (25%)	17 (18.3%)	0.162
Pregnancy, n(%)	3 (0.5%)	2 (2.2%)	0.145
Obesity, n(%)	75 (13.1%)	18 (19.4%)	0.106
Cardiac disease, n(%)	182 (31.8%)	39 (41.9%)	0.058
Chronic kidney disease, n(%)	69 (12%)	15 (16.1%)	0.271
Diabetes, n(%)	159 (27.7%)	33 (35.5%)	0.136
Immunosuppression, n(%)			
HIV**, n(%)	38 (6.6%)	7 (7.5%)	0.75
Malignancy, n(%)	35 (6.1%)	7 (7.5%)	0.602
Corticotherapy, n(%)	16 (2.8%)	2 (2.2%)	1
Others, n(%)	24 (4.7%)	4 (4.3%)	1
Vaccinated, n(%)	232 (40.5%)	26 (28%)	0.021
Subtype, n(%)			0.571
H1N1	204 (35.6%)	38 (40.9%)	
H3N2	207 (36.1%)	34 (36.6%)	
Influenza A no subtype	28 (4.9%)	5 (5.4%)	
Influenza B	134 (23.4%)	16 (17.2%)	
Cough, n(%)	500 (87.3%)	73 (78.5%)	0.024
Fever, n(%)	410 (71.6%)	66 (71%)	0.908
Myalgia, n(%)	148 (25.8%)	18 (19.4%)	0.181
Dyspnoea, n(%)	376 (65.5%)	70 (75.3%)	0.066
State of confusion, n(%)	39 (6.8%)	25 (26.9%)	< 0.001
Heart rate, median (IQR), n total	96 (83–108)	97.5 (80.5-111.5)	0.532
Systolic blood pressure [mmHg], median (IQR), n total	131 (114.5–147)	122 (108–145)	0.03
Respiratory frequency, median (IQR), n	24 (20-30.5)	30 (24.5–36)	< 0.001
Oxygen saturation [%], median (IQR), n	94 (91–96)	93 (88–96)	0.14
Oxygen saturation < 93%, n(%)	218 (38.6%)	44 (48.9%)	0.064
Horowitz Index (PaO_2_/FiO_2_^)^, median (IQR), n	280.2 (247.6-309.5)	261.9 (140-309.5)	0.001
Lymphocyte [x10^9^/L] median (IQR), n	1050 (700–1470)	810 (530–1140)	< 0.001
Hematocrit [%], median (IQR), n	39.6 (35.3–42.9)	34.8 (30.3–41.7)	< 0.001
Platelet [x109/L], median (IQR)	201,000(151,000–257,000)	202,000(154,000–260,000)	0.96
Creatinine [mg/dl], median (IQR), n	0.9 (0.7–1.1)	1 (0.8–1.8)	0.041
Urea [mg/d], median (IQR), n	36 (28–50)	53 (34–75)	< 0.001
Albumin [mg/dl], median (IQR), n	35 (32-37.8)	28.5 (24.9–32.4)	< 0.001
C-reactive protein [mg/dl], median (IQR), n	4.7 (1.9–10.9)	12.3 (4.3–23.4)	< 0.001
Pneumonia, n(%)	132 (23%)	53 (57%)	< 0.001
Pleural effusion, n (%)	15 (2.6%)	13 (14%)	< 0.001
Septic shock, n(%)	4 (0.7%)	46 (49.5%)	< 0.001
Mechanical ventilation, n(%)	9 (1.6%)	58 (62.4%)	< 0.001
ARDS***, n (%)	1 (0.2%)	10 (10.8%)	< 0.001
Coinfection, n (%)	36 (6.3%)	24 (25.8%)	< 0.001
Nosocomial infection, n (%)	56 (9.8%)	20 (21.5%)	0.001

*COPD: Chronic Obstructive Pulmonary Disease

**HIV: Human Immunodeficiency Virus

*** ARDS: Adult Respiratory Distress Syndrome

Among severe cases, 53 patients developed pneumonia (57%), whereas among non-severe cases, only 123 patients developed pneumonia (23%). Bilateral infiltrates and pleural effusions were more often found in patients with severe disease. In addition, nosocomial infections were more common in the severe case category (21.5% vs. 9.8%). A total of 76 patients were diagnosed with nosocomial influenza virus infection, and 20 of these were severe (26.3%).

Sixty patients were diagnosed with viral and bacterial coinfection, 26 developed pneumonia, and 24 had severe disease (25.8% vs. 6.3%, *p* < 0.001). The most frequent coinfection was *Streptococcus pneumoniae* (15 cases), followed by *Staphylococcus aureus* (10 cases). Thirteen patients were diagnosed with viral coinfection (4 rhinovirus, 4 coronavirus, and 5 other viruses).

The simple and adjusted effects of the main independent variables in patients with severe and non-severe disease were analyzed (Table 3).

**Table 3:** Simple and adjusted effects of the main independent variables in patients with severe/non-severe disease

	Simple regression		Backward method	
	Crude OR (95% C.I.)	sig	OR adjusted (95% C.I.)	sig
Age	0.998 (0.99–1.01)	0.776		
Smoking	1.518 (0.96–2.40)	0.074	1,625 (0,93 − 2,84)	0.088
Obesity	1.594 (0.90–2.81)	0.108		
Cardiac disease	1.548 (0.99–2.42)	0.056	1,835 (1,03–3,28)	0.040
Diabetes Mellitus	1.420 (0.89–2.25)	0.137		
Chronic kidney disease	1.405 (0.77–2.58)	0.273		
Vaccination	0.570 (0.35–0.92)	0.023	0,527 (0,28 − 0,98)	0.042
Dyspnea	1.595 (0.97–2.63)	0.068		
State of confusion at admission	5.034 (2.87–8.83)	< 0.001	4,989 (2,55 − 9,74)	< 0.001
Systolic blood pressure < 90 mmHg	3.050 (1.21–7.70)	0.018		
Horowitz index (PaO_2_/FiO_2_ < 300)	1.423 (0.83–2.44)	0.200		
Pneumonia	4.427 (2.81–6.97)	0.000	4,137 (2,39 − 7,16)	< 0.001
Oxygen saturation < 93%	1.523 (0.97–2.38)	0.065		

These analyses show that risk factors for severe disease (defined as ICU admission or death) were the development of pneumonia (OR [95% CI] = 4.14 [2.39–7.16]), history of cardiac disease (OR [95% CI] = 1.85 [1.03–3.28]), and confusion at admission (OR [95% CI] = 4.99 [2.55–9.74]). Vaccination was a protective factor (OR [95% CI]=(0.53 [0.28–0.98]).

Comparative data between the group with pneumonia and those without are presented in Table 4. Patients with pneumonia were younger (64 vs. 68 years, *p* = 0.024), with no sex or comorbidity differences. There were more pregnant women in the pneumonia group (2.2% vs. 0.2%, *p* = 0.024). The most common symptoms in patients with pneumonia were fever, myalgia, dyspnea, and confusion. These patients also had higher CRP levels (11.3 vs. 4, *p* < 0.001), a greater degree of hypoalbuminemia (32.5 vs. 35, *p* < 0.001), lower oxygen saturation (92% vs. 94%, *p* < 0.001), and lower PaO2/FiO2 (266 vs. 281, *p* < 0.001). Patients with pneumonia had a higher mortality rate, incurred more ICU admissions, experienced more mechanical ventilation, and incurred more episodes of septic shock (*p* < 0.001 in all variables).

**Table 4:** Comparative data between the group with pneumonia and those without pneumonia

	Total	No pneumonia, *N* = 481	Pneumonia, *N* = 85	*p*
Sex, (female), n(%)	328 (49.2%)	248 (51.6%)	80 (43.2%)	0.055
Age (IQR)	67.0 (53.0–77.0)	68.0 (55.0–78.0)	64.0 (49.5–75.0)	0.024
Smoking, n(%)	198 (29.7%)	134 (27.9%)	64 (34.6%)	0.088
COPD*, n(%)	160 (24.0%)	115 (23.9%)	45 (24.3%)	0.910
Pregnancy, n(%)	5 (0.8%)	1 (0.2%)	4 (2.2%)	0.023
Obesity, n(%)	93 (14.0%)	60 (12.5%)	33 (17.8%)	0.074
Cardiac disease, n(%)	221 (33.3%)	163 (34.1%)	58 (31.4%)	0.522
Chronic kidney disease, n(%)	84 (12.6%)	58 (12.1%)	26 (14.1%)	0.487
Diabetes, n(%)	193 (29.0%)	136 (28.3%)	57 (30.8%)	0.518
Immunosuppression, n(%)	136 (20.4%)	108 (22.5%)	28 (15.1%)	0.036
HIV**, n(%)	45 (6.8%)	32 (6.7%)	13 (7.0%)	0.863
Malignancy, n(%)	42 (6.3%)	32 (6.7%)	10 (5.4%)	0.553
Corticotherapy, n(%)	18 (2.7%)	17 (3.5%)	1 (0.5%)	0.033
Others, n(%)	31 (4.7%)	27 (5.6%)	4 (2.2%)	0.058
Vaccinated, n(%)	258 (38.7%)	195 (40.5%)	63 (34.1%)	0.124
Cough, n(%)	573 (86.0%)	415 (86.3%)	158 (85.4%)	0.771
Fever, n(%)	476 (71.5%)	330 (68.6%)	146 (78.9%)	0.008
Myalgia, n(%)	166 (24.9%)	109 (22.7%)	57 (30.8%)	0.029
Dyspnoea, n(%)	446 (67.0%)	309 (64.2%)	137 (74.1%)	0.016
State of confusion, n(%)	64 (9.6%)	37 (7.7%)	27 (14.6%)	0.007
Season 12–13	51 (7.7%)	35 (7.3%)	16 (8.6%)	0.472
Season 13–14	116 (17.4%)	78 (16.2%)	38 (20.5%)	
Season 14–15	248 (37.2%)	185 (38.5%)	63 (34.1%)	
Season 15–16	251 (37.7%)	183 (38.0%)	68 (36.8%)	
Subtype, n(%). H1N1	242 (36.3%)	161 (33.5%)	81 (43.8%)	0.066
H3N2	241 (36.2%)	186 (38.7%)	55 (29.7%)	
Influenza A no subtype	33 (5.0%)	23 (4.8%)	10 (5.4%)	
Influenza B	150 (22.5%)	111 (23.1%)	39 (21.1%)	
Heart rate, median (IQR), n total	96.0 (82.0–109.0)	95.0 (82.0–108.0)	98.5 (82.3–112.8)	0.062
Systolic blood pressure [mmHg], median (IQR), n total	131.0 (113.0–147.0)	131.0 (114.3–146.0)	130.0 (110.0–149.0)	0.557
Respiratory frequency, median (IQR), n	26.0 (20.0–32.0)	26.0 (20.0–32.0)	25.0 (20.0–32.0)	0.880
Oxygen saturation [%], median (IQR), n	93.0 (90.0–96.0)	94.0 (91.0–96.0)	92.0 (88.0–95.0)	< 0.001
Oxygen saturation < 93%, n(%)	262 (40%)	170 (35,8%)	92 (51,1%)	< 0.001
Horowitz Index (PaO_2_/FiO_2_^)^, median (IQR), n	276.2 (242.9–309.5)	281.0 (250.0–314.3)	266.7 (223.8–304.8)	< 0.001
Alveolar-arterial gradient, median (IQR), n	46.0 (38.0–55.5)	45.0 (36.9–53.8)	49.2 (41.0–59.0)	< 0.001
Alveolar-arterial gradient adjusted for age, median (IQR), n	17.4 (14.3–19.7)	17.4 (14.7–19.7)	17.2 (13-19.9)	0.003
Lymphocyte [x10^9^/L] median (IQR), n	1000.0 (670.0–1447.5)	1000.0 (670.0–1407.5)	980.0 (662.5–1520.0)	0.732
Hematocrit [%], median (IQR), n	39.1 (34.7–42.7)	39.2 (34.8–43.0)	38.8 (34.1–42.4)	0.355
Platelet [x109/L], median (IQR)	201,500 (151,000–258,250)	201,000(148,000–253,000)	202,000 (159,500–276,500)	0.196
Creatinine [mg/dl], median (IQR), n	0.9 (0.7–1.1)	0.9 (0.7–1.1)	0.9 (0.7–1.3)	0.251
Urea [mg/d], median (IQR), n	36 (28–53)	36 (28–52)	38 (29–65.5)	0.096
Albumin [mg/dl], median (IQR), n	34.6 (31- 37.3)	35 (31.9–37.9)	32.5 (29.2–36.4)	< 0.001
C-reactive protein [mg/dl], median (IQR), n	5.1 (2.0–11.9)	4 (1.6–9)	11.3 (4.7–22)	< 0.001
Pleural effusion, n (%)	28 (4.2%)	16 (3.3%)	12 (6.5%)	0.069
Septic shock, n(%)	50 (7.5%)	16 (3.3%)	34 (18.4%)	< 0.001
Mechanical ventilation, n(%)	67 (10.1%)	22 (4.6%)	45 (24.3%)	< 0.001
Intensive care unit admission, n(%)	73 (11.0%)	28 (5.8%)	45 (24.3%)	< 0.001
Death, n(%)	39 (5.9%)	17 (3.5%)	22 (11.9%)	< 0.001
Bacterial coinfection, n(%)	60 (9.0%)	34 (7.1%)	26 (14.1%)	0.005
Nosocomial infection, n(%)	76 (11.4%)	66 (13.7%)	10 (5.4%)	0.003

*COPD: Chronic Obstructive Pulmonary Disease. HIV**: Human Immunodeficiency Virus

In multivariate analysis, variables associated with the development of pneumonia were oxygen saturation (OR [95% CI] = 1.05 [1.00-1.11]) and CRP (OR [95% CI] = 1.71 [1.05–1.11]). The remainder of the analysis is presented in Table 5.

**Table 5:** Simple and adjusted effects of the main independent variables in patients with pneumonia

	Simple regression		Backward method	
	Crude OD (95% C.I.)	Sig.	OR adjusted (95% C.I.)	Sig.
Female sex	0.716 (0.51–1.01)	0.055	0.644 (0.38–1.09)	0.099
Age	0.990 (0.98–1.00)	0.046	0.984 (0.97–1.00)	0.054
Fever	1.713 (1.15–2.56)	0.009		
Myalgias	1.520 (1.04–2.32)	0.030	1.701 (0.95–3.05)	0.074
Dyspnoea	1.589 (1.09–2.32)	0.016		
State of confusion	2.051 (1.21–3.48)	0.008	2.094 (0.92–4.76)	0.077
Heart Rate	1.011 (1.00–1.02)	0.012		
Oxygen saturation [%]	1.065 (1.03–1.10)	0.000	1.052 (1.00-1.11)	0.049
Horowitz Index (PaO_2_/FiO_2)_	0.995 (0.99–1.00)	0.000		
Urea [mg/dl]	1.008 (1.00–1.01)	0.010		
C-reactive protein [mg/d]	1.066 (1.04–1.09)	0.000	1.078 (1.05–1.11)	0.000
Albumin [mg/dl]	0.919 (0.88–0.96)	0.000		

## Discussion

This study involved 666 patients admitted to the hospitalization ward with a laboratory-confirmed influenza virus infection, 14% of whom presented with severe disease (11% required intensive care and 6% died). Risk factors related to severe infection (defined as death or ICU admission) at the time of hospital admission were pneumonia, confusion at admission, and cardiac disease. Influenza virus vaccination was found to be a protective factor against ICU admission and death. In contrast, low oxygen saturation and high levels of CRP at admission were associated with an increased risk of pneumonia development in patients admitted with influenza virus infection.

Most studies have analyzed complications related to influenza in ICU situations while few studies have analyzed these complications in hospital wards. In a French prospective multicenter study, Loubet et al. found a similar proportion of pneumonia at 30%, 15% of the total requird admission to the ICU and 4% of the total died; age over 65 years was the only risk factor for severe cases [9]. Other studies reported higher rates of admission to the ICU of approximately 25–30% with a mortality rate of approximately 13% [10, 11]. Contrary to other studies, we did not find differences in age, sex, or influenza subtype between severe and non-severe cases [12]. In a multicenter Spanish study of 1,726 patients where influenza was subtyped, the mortality rate was higher in patients aged > 75 years and in patients 65–74 years, although they were admitted to the ICU less often, and subtype H1N1 was associated with more intensive care requirements [10]. The association of severity with heart disease has been described in other studies with similar populations, however, without reaching statistical significance [9]. Many studies have related recent influenza virus infection with a subsequent onset of ischemic heart disease after admission [13]. However, we analyzed but did not find statistically significant differences between other comorbidities such as COPD, diabetes, and chronic renal diseases as has been found in other studies [11–13]. We did not find differences in terms of immunosuppression perhaps because few immunocompromised patients were included in our study compared with other studies [10, 14]. Seasonal influenza vaccination has been shown to be a protective factor against complications and reduces hospital admissions [15–17]. Few studies have analyzed the efficacy of seasonal vaccination in similar populations. In a study by Loubet et al., 38% of patients were vaccinated, and vaccination was not related to complications or death; however, it was a protective factor regarding admission to the ICU [9]. A Spanish case-control study by Castilla et al. showed that influenza vaccine protects more in outpatient cases (75%) and severe cases -death or admission to the ICU- (89%), but not so much in non-severe hospitalized cases (60%) [15].

In our study, 30% of admissions with influenza infection presented with pneumonia, which was also an independent risk factor for severity. In previous studies, pneumonia is a complication in up to a third of cases of influenza and is also an independent risk factor for death [16]. In a Spanish study by Viasus et al., factors associated with the severity of patients with pneumonia due to all causes were analyzed, and influenza A (H1N1)pdm09 and Influenza B were significant, as well as the presenceof Sat O2 < 90% and the presence of confusion. One of the aims of their study was to be able to apply the Bewick Score to differentiate influenza A pneumonia from the rest of the causes [17, 18]. In our study, sufficient consistency in the multivariate analysis did not exist to create a predictive score with optimal levels of adjustment and explanatory capacity.

Difficulties in differentiating between pneumonia of primary viral origin and viral pneumonia with bacterial superinfection persist. There is great variability in the symptoms of patients with viral infection; moreover, viral presence in the upper respiratory tract does not imply that the virus is the cause of pneumonia [2]. A study in 1957 by Louria et al. showed that the same virus could produce a primary viral pneumonia, pneumonia with bacterial superinfection, and concomitant or mixed viral and bacterial pneumonia, depending on the presentation of symptoms and radiologic findings [19]. Subsequent studies showed how the elevation of inflammatory markers, such as CRP and procalcitonin, helped to differentiate bacterial from viral pneumonia, including bacterial and viral infection pneumonia [5, 20]. In most studies on pneumonia with bacterial coinfection, elevated CRP and procalcitonin levels have been observed [21]. In the study by Bello et al., procalcitonin levels were higher in the group of bacterial and mixed pneumonias, while CRP was higher only in the mixed pneumonias [5]. In our study, patients with severe disease had a median CRP of 12.3 mg/dl in comparison with 4.7 mg/dl in the non-severe category (*p* < 0.001), and a significant association was observed between levels of CRP and the development of pneumonia. However, we did not differentiate between viral and bacterial origin due to classification difficulties as it was a retrospective study. Viral infections often cause a decrease in procalcitonin levels [20, 22]. Recent studies involving admitted patients with COVID-19 also showed the utility of CRP and procalcitonin analyses to help differentiate cases of pure viral pneumonia from secondary bacterial infection [23, 24].

Regarding the coinfections observed in this study population, the results regarding isolated microorganisms were very similar to those of other studies; *Pneumococcus* sp. was found most frequently, followed by *S. aureus*, and rhinovirus was the most isolated virus for viral coinfection [1, 11, 17, 25, 26].

### Limitations of the study

This was a retrospective single-center study whereby available data were limited and based on a review of the clinical history. As such, some risk factors, such as obesity, may have been underreported. Moreover, some quantitative variables, such as respiratory rate, may have been underrepresented. The improvement in diagnostic capability of influenza infection over the four-year study period presents a possible bias, because there were fewer patients in the first few years but the cases were more severe, and there were more cases in the latter years. The lack of differences seen in patients with other comorbidities or by age could be due to patient selection bias, in that it is likely that younger patients were less frequently admitted to the hospital, and older patients with comorbidities were less frequently admitted to the ICU. This is supported by the observation that there were 20 patients who died and were not admitted to the ICU, and also possibly related to the protective effect of the vaccine in the older patients with more comorbidities due to having a higher proportion of vaccination coverage. Finally, procalcitonin data were not available to differentiate between viral and bacterial infection, which could have helped to determine final diagnoses.

## Conclusions

Various factors that can be detected upon arrival of patients admitted for influenza virus that are associated with poor prognosis (death and admission to ICU) were pneumonia, state of confusion, and history of cardiac disease. Influenza vaccination was found to be a protective factor. Additionally, higher levels of CRP and lower levels of oxygen saturation could predict the development of pneumonia in these patients. Pneumonia appears as a complication in one-third of patients with influenza virus, and despite its frequency, it is still difficult to classify as primary viral pneumonia or secondary bacterial superinfection; therefore, more studies are needed to establish diagnostic tools that help to direct more targeted treatment and improve prognosis.

## Data Availability

The datasets generated and analyzed during te current study are not publicly available due the lack of informed consent and ethics approval to share, but are available from the corresponding author on reasonable request.

## Declarations

All the methods were carried out in accordance with relevant guidelines and regulations.

## Abbreviations

ICU: Intensive Care Unit
CRP: C-reactive protein
OR: Odds Ratio
PCR: Polymerase Chain Reaction
COPD: Chonic Obstructive Pulmonary Disease
PaO_2_/FiO_2_: Arterial oxygen partial pressure to fractional inspired oxygen ratio (or Horowitz index)
